# Lessons learned from two applied approaches for treatment of aged DDT-contaminated soil with white-rot fungi

**DOI:** 10.1371/journal.pone.0357404

**Published:** 2026-09-11

**Authors:** Stephanie Casey, Karin Wiberg, Anna-Karin Dahlberg, Malin Hultberg, Anja Enell, Yevheniya Volchko

**Affiliations:** 1 Department of Aquatic Sciences and Assessment, Swedish University of Agricultural Sciences (SLU), Uppsala, Sweden; 2 IVL Swedish Environmental Research Institute, Stockholm, Sweden; 3 Department of Biosystems and Technology, Swedish University of Agricultural Sciences, Alnarp, Sweden; 4 Swedish Geotechnical Institute (SGI), Linköping, Sweden; 5 Division of Geology and Geotechnics, Department of Architecture and Civil Engineering, Chalmers University of Technology, Gothenburg, Sweden; Ataturk University: Ataturk Universitesi, TÜRKIYE

## Abstract

The persistence of dichlorodiphenyltrichloroethane (DDT) in soil poses a serious environmental challenge due to its toxicity, hydrophobicity, and resistance to degradation. White-rot fungi, such as *Pleurotus ostreatus*, show potential for bioremediation of persistent organic pollutants via ligninolytic enzymes. The aim of this study was to evaluate whether *P. ostreatus* spent mushroom substrate (SMS), used directly as a waste product from mushroom production and without pre-conditioning, could be applied to aged DDT-contaminated soil and result in measurable reductions in DDT and its transformation products (collectively referred to as DDX) in a pot-scale mesocosm experiment designed to mimic field application. We further evaluated whether pre-inoculated fungal substrates introduced into contaminated field soil could establish in the amended soil and be associated with measurable reductions in DDX under environmentally realistic conditions. Under the conditions tested, no measurable degradation of DDT or its transformation products was observed in either the pot experiment or the field trial. Considering that significant reduction in DDX has been observed in similar lab experiments with DDT-spiked soils elsewhere, the findings in this study highlight critical limitations to mycoremediation of aged DDT in soils under laboratory and field conditions tested in this study, particularly fungal viability, substrate quality, and soil colonization constraints. Rather than demonstrating treatment efficacy, this study identifies and outlines constraints that limited performance and provides transferable insights for the future development of white-rot fungal remediation strategies for aged DDT-contaminated soils under environmentally realistic conditions. It emphasizes the need to enhance fungal activity and enzyme production, for example through pre-conditioning of SMS, while also addressing the limited bioavailability of DDX in historically contaminated soils, which may constrain fungal access to the contaminants and reduce treatment efficacy in real-world settings.

## 1. Introduction

Dichlorodiphenyltrichloroethane (DDT) is an insecticide. DDT’s chemical structure, containing five chlorines and two aromatic rings, makes it highly hydrophobic (log *K*_*OW*_ *p,p*’-DDT: 5.92, according to ARCHem SPARC 2010 [1] with strong sorption to soil organic matter. This reduces its bioavailability but also protects it from degradation, allowing it to persist for decades [2]. When degradation does occur, it often results in persistent transformation products such as dichlorodiphenyldichloroethylene (DDE), dichlorodiphenyldichloroethane (DDD), and dicofol, which remain toxic environmental pollutants. These compounds all have high hydrophobicity, with log *K*_*OW*_ values between 6.0 and 6.9 [1], and yet still pose a risk of leaching should they become mobile, contaminating groundwater and aquatic ecosystems. DDT exposure in wildlife leads to bioaccumulation and biomagnification, disrupting their endocrine systems and reducing reproductive success [3]. In humans, DDT is classified as a probable carcinogen [3].

DDT has been used since 1939 worldwide, playing a crucial role in controlling disease vectors such as mosquitoes that transmit malaria and typhoid. However, its persistence and toxicity have led to severe environmental and health consequences. Concerns over its effects on wildlife and human health were first highlighted in the 1960s, notably in Rachel Carson’s *Silent Spring* [4]), prompting regulatory action. A global ban on agricultural use followed in 2004 under the Stockholm Convention on Persistent Organic Pollutants [5], with Sweden being the first country to ban its use in 1969 [6]. Despite these restrictions, DDT remains in use for vector control in some regions and persists at high concentrations in soils worldwide, particularly in former agricultural areas. In Sweden alone, very large areas of land across ca 3700 plant nursery sites remain contaminated with DDT, posing risks to local ecosystems and, in some cases, human health, and thus require detoxification and restoration [7–9]). Conventional *ex-situ* remediation methods, mainly excavation for disposal in landfill, destroy soil functions, produce considerable waste, and result in high costs to society [10]. In contrast, bioremediation offers a low-input and low-impact approach to degrading DDT in contaminated soils, with fungi playing a particularly important role. White-rot fungi, such as *Pleurotus ostreatus*, have been widely studied for their ability to degrade persistent organic pollutants due to their production of oxidative enzymes, including laccases, manganese peroxidases (MnP) and other peroxidases [11]. These enzymes, originally evolved for lignin degradation, are capable of breaking down structurally complex pollutants like DDT [12].

One potential large-scale and cost-effective approach to fungal bioremediation (mycoremediation) involves the use of spent mushroom substrate (SMS) – the residual organic material left after mushroom cultivation, which still contains active fungal biomass. The mushroom industry produces approximately 5–6 kg of SMS for every kg of mushrooms, and 27% of commercially produced mushrooms world-wide are oyster mushrooms (*P. ostreatus*), amounting to approximately 1.7 million tonnes yearly [13,14]. The SMS is often treated as waste but has also been explored as a resource in different settings [15]. Numerous studies have explored SMS in various remediation contexts including for treatment of PAHs, pesticides and heavy metals [16]. For example, SMS of *Pleurotus pulmonarius* was used to degrade pentachlorophenol in contaminated water with up to 89% efficiency in its removal [17]. Similarly, laccase activity in *P. eryngii* SMS was comparable to pre-fruiting levels, suggesting its viability bioremediation [18]. For many organic contaminants, remediation effects have been linked to the ligninolytic enzymes and cytochrome P450 systems to a large extent [19]. There is also research showing that indigenous microbial communities were found to be capable of degrading DDT, DDE and DDD in aged, contaminated soils [20], and that the stimulating effect of adding a nutrient rich substrate, such as SMS, may further enhance this process [21].

*P. ostreatus* spent mushroom substrate has previously been shown to degrade DDT under laboratory conditions, including in artificially DDT-contaminated soil [12]. However, this was achieved using a very high amendment ratio of 1:1 on a dry-weight basis, i.e., an SMS addition exceeding the soil mass, which has limited relevance for large-scale applications. The aim of this study was to evaluate whether *P. ostreatus* spent mushroom substrate (SMS), reused directly and without pre-treatment from mushroom production, could function as a circular, waste-derived remediation amendment for aged DDT-contaminated soil under mesocosm conditions designed to mimic in-situ field application at a large scale. We further aimed to evaluate whether pre-inoculated fungal substrates introduced into contaminated field soil could establish in the amended soil and be associated with measurable reductions in DDX under environmentally realistic conditions.

To address this, we evaluated the potential for fungal bioremediation of DDT-contaminated soils collected from a historically polluted forest nursery site in southern Sweden. Two complementary strategies were tested: a controlled mesocosm (pot-scale) experiment using *P. ostreatus* SMS, and an *in-situ* field trial applying spawn of *P. ostreatus* and *Stropharia rugosoannulata* pre-inoculated on straw. Both approaches relate to commercial mushroom production practices, highlighting the possibility of turning organic waste into a valuable resource for remediation. Additionally, these trials were designed to assess the practical challenges of implementing fungal-based remediation under natural conditions, including fluctuating soil moisture, heterogeneity, and interactions with native microbiota.

## 2. Methods

### 2.1 DDX-contaminated soil for experiments

The 23-hectare Kolleberga forest nursery, located in the southern region of Sweden (Klippan municipality, coordinates: 56.070773, 13.261842), served as the source of historically DDT-contaminated soil utilised in both laboratory and field experiments. DDX contamination at this location, as in many other sites with a similar contamination problem, is limited to a shallow depth of 30 cm and presents risks to organisms higher up in the food chain [22]. The land is no longer used for productive forestry; instead, it is maintained by planting a mix of grasses, which are regularly cut and then ploughed back into the soil. The underlying soil consists of a glacio-fluvial sediment of loamy sand.

For the mesocosm experiment, a total of 55 kg of DDT contaminated soil was collected from surface soil (0–20 cm depth) at the Kolleberga site in April 2023. The soil was sieved through a 4 mm mesh in the laboratory to remove larger debris and subsequently stored at 4 °C in the dark for one week prior to setting up the pot-scale experiment. Soil subsamples of 5 g wet weight (ww) were taken for soil characterization, and properties are shown in S1 Table in S1 File, with method details also provided. Water content was measured gravimetrically by drying at 105 °C (24 h), and organic matter content was measured by combustion at 505 °C for 4 h (S1 Table in S1 File). The mesocosm experiment set-up is summarized in Section 2.2. A designated area approximately 10 x 7 m within the Kolleberga site was selected for the field experiment. Information about the field trial set-up is provided in Section 2.3.

### 2.2 SMS mesocosm experiment

#### 2.2.1 *P. ostreatus* SMS.

A total of 10 kg of *P. ostreatus* SMS was collected from the commercial mushroom producer Fungigården AB (Harplinge, Sweden) and stored at 4 °C in the dark for one week prior experimental start. The SMS had a C:N ratio of 60 ± 6 and a water content of 64 ± 3%. Before the experiment, the SMS was thoroughly mixed manually. A composite SMS sample in triplicate was collected to analyze oxidative enzyme activities. The SMS was used without pre-conditioning to reflect realistic conditions in which such materials may be applied directly as a waste product from mushroom production to large DDT-contaminated areas. Fresh *P. ostreatus* SMS from Fungigården AB (Harplinge), collected after a single mushroom harvest, was used directly in this experiment after one-week cold storage (+7°C). It appeared moist and fresh, with visible white hyphal growth.

#### 2.2.2 Experimental set-up.

A portion of the manually mixed SMS (3 kg) was sterilized by autoclaving at 121 °C for 2 h (water-steam cycle). A composite subsample of this autoclaved SMS (10 g) in 3 replicates was analyzed for loss of ligninolytic activity, which was confirmed by enzyme assay (Section 2.5). Wet straw was prepared by soaking 90 g of dry straw pellets in 360 g of 60 ºC water.

A total of 54 kg of soil (ww) was homogenized, and divided into 12 equal portions, with each portion (4.5 kg) placed into a zinc pot cleaned with ethanol and water. The pots had a capacity of 27 L (dimensions: 24 x 37 x 24 cm) and were obtained from Biltema AB (Sweden). The experiment was composed of two treatments, one control and one blank with three replicates of each (Table 1). In treatment 1, the soil was mixed with 900 g ww of SMS (soil:SMS 10:2 weight/weight (w/w)). In treatment 2, the soil was mixed with 900 g SMS and 450 g of wet straw (soil:SMS:straw 10:2:1 w/w). In the control, the soil was mixed with 900 g autoclaved SMS (soil:autoclaved SMS 10:2 w/w) with no enzymatic activity. The target volumetric ratio of SMS to soil was 20%. The experimental blank consisted of untreated soil without any amendments and therefore represents a true control for natural attenuation processes..

**Table 1:** The mesocosm experimental set-up (n = 3).

Treatment	Soil (g) (ww)	Wet straw (g) (ww)	Fresh SMS (g) (ww)	Autoclaved SMS (g) (ww)
Treatment 1	4 500	0	900	0
Treatment 2	4 500	450	900	0
Control	4 500	0	0	900
Experimental blank	4 500	0	0	0

SMS: spent mushroom substrate (fresh) of *P. ostreatus*; ww: wet weight.

During the experiment, the pots were kept in darkness at 21ºC and thoroughly mixed manually, with moisture content adjusted to ~60% of water holding capacity (WHC, ISO 11268–2:2012), every third day for 4 months. Photos of mesocosms are shown in S1 Fig.

#### 2.2.3 Experimental sampling.

Three cores taken from each pot were combined into one composite sample (100 g) per pot after substrate addition on day 1 (T0) and after 4 months (T1). Before sampling, the soil in each pot was thoroughly mixed. Each soil sample was divided into two portions: one for oxidative enzyme analysis (50 g) and one for DDX concentrations analysis (50 g) (Section 2.4). The latter samples were, after being sieved (2 mm), placed in amber glass jars with PTFE-lined lids, and stored at −20°C until chemical analysis. All samples were analysed after termination of the experiment. The pots were weighed at each sampling time point to assess weight loss due to mineralisation of organic carbon, and water loss. No fruiting bodies were observed.

### 2.3 Field-scale experiment with pre-inoculated substrate

#### 2.3.1 Spawn and substrate preparation.

Two fungal species were selected: *P. ostreatus* strain M2191, grown on wheat grain spawn, and *Stropharia rugosoannulata* strain M5012 grown on straw spawn. Both straw and wheat were sterilised by autoclave and mixed with 2% CaCO_3_, as per standard cultivation practise. *S. rugosoannulata* was included as a second fungus to evaluate as this species has been shown to degrade straw in fields using ligninolytic enzymes [23]. Thus, this fungus has demonstrated its capability for survival and growth in soil settings. Both fungal species were obtained from the company Mycelia BVBA (Deinze, Belgium). The inoculum for field application consisted of 30 L of fungal spawn mixed with 13 kg of heat-treated straw pellets and 25 L of water, creating ~60 L of pre-inoculated substrate per treatment. This mixture was incubated in composting buckets at the field site Kolleberga for 7 days prior to mixing into the soil. Fungal growth could be observed visually (S2A and S2B Fig) before inoculation of the mixture.

#### 2.3.2 Experimental set-up.

**Fig 1:**
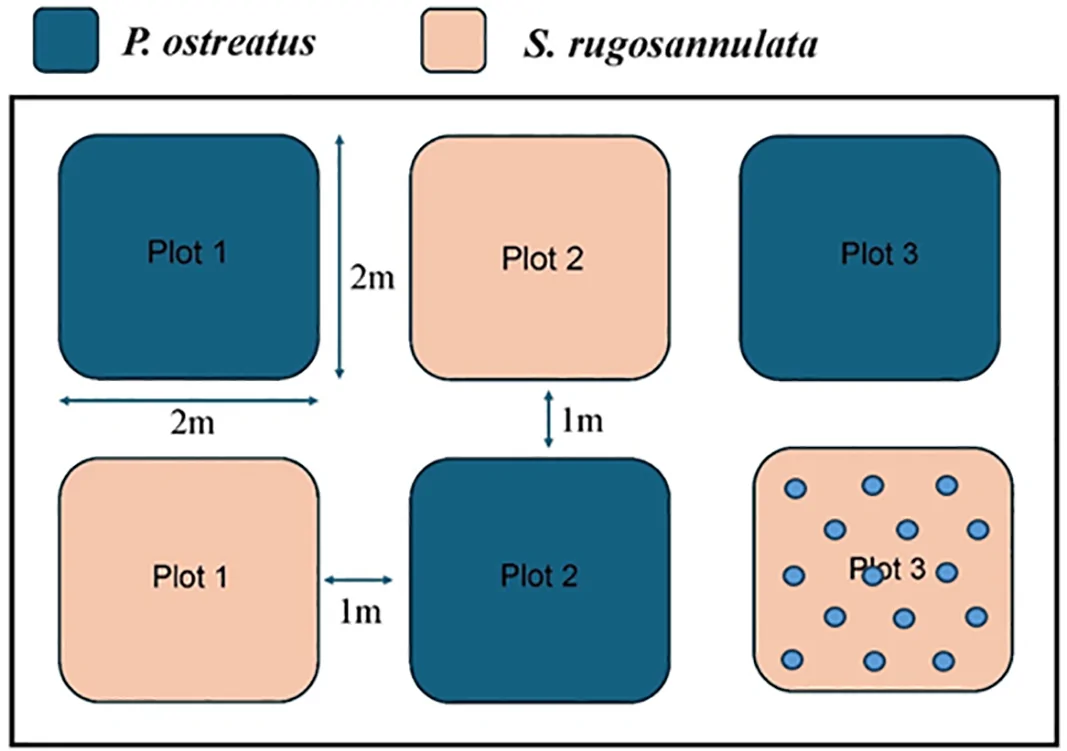
Schematic of experimental plot area at Kolleberga plant nursery (Klippan municipality, Sweden) with sampling grid system used for all plots shown on S. rugosannulata Plot 3.

Six plots (2 × 2 m each) were established in a randomized block design with 1 m spacing between them within the Kolleberga site (Fig 1). Each plot was excavated to a depth of ~40 cm, corresponding to a DDX-contaminated soil volume of ~1 600 L. To isolate the test soil and prevent root infiltration while allowing water flow, permeable weed barrier fabric was installed in each excavated area. To ensure consistent baseline conditions across all treatments, all excavated soil was combined and thoroughly homogenized using a mechanical excavator before being returned to the plots. Each 2 × 2 m plot received one of the two treatments (*P. ostreatus*, *S. rugosoannulata*), with three replicate plots per treatment. The pre-inoculated substrate (20 L per plot) was spread evenly across the surface and tilled into the upper 10–15 cm of soil (~400 L) using handheld tilling tools. This depth was selected due to the behaviour of DDX remaining mostly in the top 20 cm of the soil profile, and because the conditions below 20 cm are less favorable for fungal growth. The target volumetric ratio of spawn to soil was approximately 5% (v/v) (spawn:soil:substrate ≈ 0.5:8:1.5 v/v). Photos of the field site are shown in S2C and S2D Fig.

The duration of the field experiment (6 weeks) was selected to capture early-stage fungal establishment and initial responses under environmentally realistic conditions. Given the exploratory nature of this study and the focus on feasibility in field conditions, the experimental timeframe was designed to assess whether fungal colonization and associated processes could be initiated within a short-term monitoring period.

#### 2.3.3 Sampling of soil and fruiting bodies.

**Fig 2:**
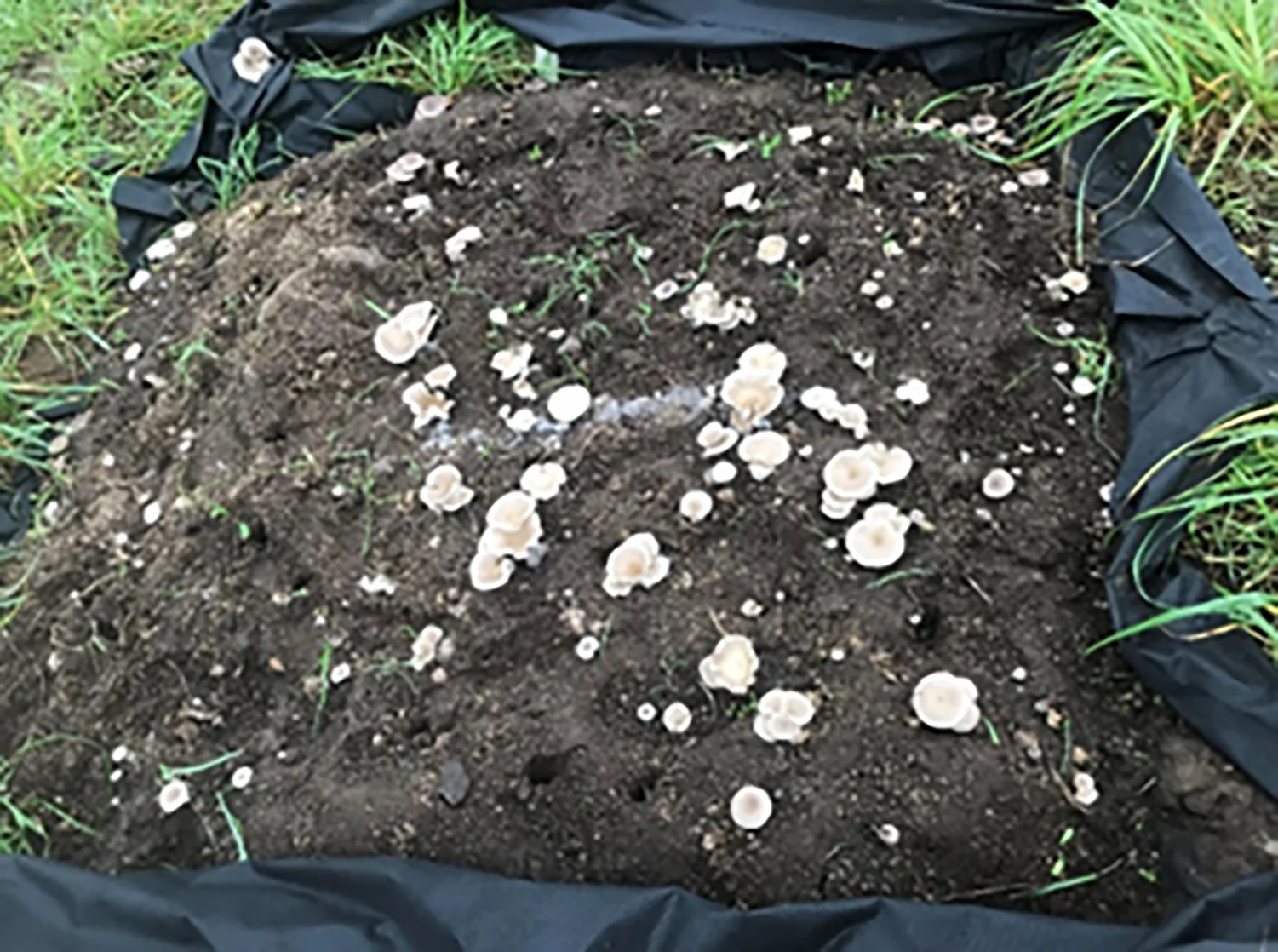
Fruiting bodies of P. ostreatus emerged after five weeks in all P. ostreatus treated plots at Kolleberga plant nursery.

Soil sampling was conducted at T0 (immediately after inoculation) and T1 (6 weeks after inoculation). At each time point, twelve soil cores (15 cm depth) were collected from each plot in a grid system (Fig 1) using a soil corer, rinsed with ethanol and water between sampling, and pooled to form a composite sample. Samples were sieved (2 mm), placed in amber glass jars with PTFE-lined lids, and frozen at −20°C within 6 hours of collection. After 5 weeks, fruiting bodies appeared in each of the 3 plots of *P. ostreatus* treatment (Fig 2). The mushrooms were carefully sampled, brushed clear of soil, rinsed with water and frozen at −20°C until analysis.

### 2.4 Chemical analysis

The target analytes included the parent compounds *p,p*’-and *o,p’*-DDT and their breakdown products *p,p*’-and *o,p’*-DDE (1,1-dichloro-2-bis(p-chlorophenyl)ethene), *p,p*’-and *o,p’*-DDD (1,1-dichloro-2,2-bis(p-chlorophenyl)ethane), *p,p*’-DDM (2,2-bis(chlorophenyl)methane), *p,p*’-DDMU (1-chloro-2,2-bis(p-chlorophenyl)ethene), *p,p*’-DBP (4,4’-dichlorobenzophenone) and dicofol. Vendors for all chemicals and materials used in analysis are provided in S2 Table in S1 File.

#### 2.4.1 DDX in soil and fruit bodies – Mesocosm and field experiments.

Prior to extraction, the soil and fruiting body samples were freeze dried using a LyoDry Compact Benchtop Freeze Dryer (Mechatech systems, UK) at −55 °C and 7.5E^-2^ mbar. Freeze-dried soil and fruit body samples (0.5–1 g) were weighed into Soxhlet thimbles (pre-cleaned with dichloromethane) for extraction. Each sample was spiked with 10 μL of the ^13^C-labelled internal standard mixture (10 ng; S3 Table in S1 File) and extracted with 200 mL of dichloromethane (DCM) for 24 h following Soxhlet extraction protocols similar to Huang et al. [24]. The extracts were concentrated to 5 mL by rotary evaporation and further reduced under nitrogen before solvent exchange to *n*-hexane (final volume 1 mL). Procedural solvent blanks were included in each batch for quality assurance, though within the mesocosm study they were spiked immediately before analysis.

Sulphur removal was carried out by adding 1 g of activated granular copper and thoroughly mixing. For further purification, the extracts were passed through alumina-silica columns (10 mm diameter) packed with 6.1 g of Al_2_O_3_:SiO_2_ (1:2 v/v) and topped with 3 g of sodium sulphate [24]. Target compounds were eluted using 45 mL of a DCM:*n*-hexane mixture (2:3 v/v). The solvent was again concentrated and exchanged to isooctane, with a final volume of 1 mL. A recovery standard (10 ng; S3 Table in S1 File) was added, and the sample was vortexed (5 min) before transferring an aliquot into GC vials for instrumental analysis.

#### 2.4.2 Instrumental analysis of DDX.

Compound analysis of soil, and fruit body extracts was performed using an Agilent 7890A gas chromatograph coupled with an Agilent 7010 triple quadrupole mass spectrometer (GC-MS/MS), equipped with an Agilent 7693 autosampler for automated injection. Injection volume was 2 μL. Samples were injected in splitless mode at 275 °C using a Siltek®-deactivated gooseneck liner (Restek, USA) to enhance transfer and minimize decomposition. Compound separation was achieved on a DB-5 capillary column (60 m × 250 µm i.d., 0.25 µm film thickness; Agilent Technologies). The GC oven program was as follows: initial temperature at 100 °C (1 min hold), ramped at 15 °C min^−1^ to 250 °C, then at 10 °C min^−1^ to 280 °C (held for 12 min), followed by 20 °C min^−1^ to 300 °C (held for 3 min). Helium (He) was the carrier gas at a constant flow of 2 mL min^−1^. The MS was operated in electron ionization (EI) mode at 70 eV, with ion source and transfer line temperatures set to 300 °C and 310 °C, respectively. The system operated under multiple reaction monitoring (MRM) conditions. Precursor ions were isolated in Q1, fragmented in Q2 using nitrogen as the collision gas (helium as quench gas), and product ions monitored in Q3. Data acquisition and quantification were performed using MassHunter Quantitative Analysis software (Agilent).

#### 2.4.3 Quality assurance and quality control.

A *p,p’*-DDT solution of 250 ng L^-1^ was injected separately to monitor in-injector degradation of DDT to DDD during the GC-analysis. The degradation remained below 10% for all samples (S4 Table in S1 File), meeting the threshold accepted as per Budde et al. [25], and as such the compounds are reported separately.

The limit of detection (LOD) was set to a S/N ratio of ≥3. The limit of quantification (LOQ) was defined as the lowest calibration solution that could be reliably quantified by the instrument and fulfilling a S/N criterion of ≥10. However, if contamination was present in the blanks, the LOQ was defined as follows:


$$\begin{array}{c} \text{LOQ }=\text{ average procedural blanks }+(\text{10 x stdev procedural blanks}). \end{array}$$

In cases when the target analyte was < LOD or <LOQ, the data point was excluded from reporting and replacement values were used for statistical purposes only, as described in Section 2.6. The only results affected of blank levels were 3 values for *p,p’*-DDM in the pot-scale mesocosm experiment.

Any individual samples that exceeded 200% recovery, or were below 50%, were excluded (indicated in S5–S7 Tables in S1 File). The recovery of dicofol was below our acceptable threshold of 50% for the results to be considered valid in the mesocosm experiment, and *o,p’*-DDE recoveries were below the 50% threshold in the mesocosm experiment and the fruit body analysis. These compounds were therefore excluded from further evaluations. Remaining recoveries had mean ± standard deviation of 95% ± 26% for soil samples from the mesocosm experiment, 106% ± 18% in soil samples from the field experiment and 120% ± 15% for fruit body samples. The low recovery of dicofol in the mesocosm experiment may be attributed to degradation of dicofol to *p,p*’-DBP during the analysis (Yin et al., 2017) [26].

### 2.5 Enzyme activity assay – SMS mesocosm experiment

Ligninolytic enzyme activity of subsamples from homogenised composite samples were assessed in the mesocosm experiment using the MBTH-DMAB colorimetric method [27,28]. Absorbance at 590 nm, resulting from the oxidative coupling reaction, was recorded every 15 seconds for a duration of 10 min using a SpectraMax Plus 384 microplate reader (Molecular Devices, USA). Enzyme activity was calculated based on the initial rate of absorbance increase, referenced against a standard curve prepared using purified MnP (Sigma-Aldrich, product no: 803057; CAS: 114995-15-2) and expressed as U g^−1^.

### 2.6 Statistical analysis

All statistical analyses were conducted in RStudio (version 2025.09.2, Build 418).

For each compound, a linear mixed-effects model was fitted using the lmer function from the lme4 package, with Amount as the response variable and Time_Point, Treatment, and their interaction as fixed effects. Replicate (a–c) was included as a random intercept to account for the paired structure of the dataset and repeated measures across time. Replacement values for statistical evaluation were calculated as (mean(blank) + 3·SD(blank))/2 for LOD, and (mean(blank) + 10·SD(blank))/2 for LOQ. Replacement values were used when S/N < 3 (LOD) or S/N < 10 (LOQ).

Model assumptions were evaluated for each compound by inspection of residual diagnostic plots. Residual normality was assessed using the Shapiro–Wilk test, and homoscedasticity of residuals was evaluated using the Breusch–Pagan test. Log-transformation was not required.

Estimated marginal means (EMMs) were calculated using the emmeans package to quantify differences between Time_Point 0 and Time_Point 1 within each Treatment × Compound combination. Pairwise contrasts were corrected for multiple comparisons using the false discovery rate (FDR) method. Effect sizes (estimated change from Time_Point 0–1) and associated adjusted p-values were extracted to summarise direction and significance of temporal change for each compound and treatment.

To evaluate whether overall chemical profiles changed as a function of Treatment, Time_Point, or their interaction, a multivariate analysis of variance (PERMANOVA) was performed using the adonis2 function from the vegan package. A compound-by-sample matrix was constructed, and Euclidean distances were used as the dissimilarity metric. PERMANOVA tests were run with 999 permutations, with Time_Point, Treatment, and their interaction included as predictors.

Total compound effects were also analysed by summing all compound concentrations for each sample and modelling these totals using the same mixed-effects structure described above (Time_Point × Treatment with Replicate as a random effect). EMMs were used to assess temporal changes in total concentration within treatments.

Due to the limited number of replicates (*n* = 3), the statistical power of the analyses was constrained, and results should be interpreted with caution.

### 2.7 Ethics statement

This study did not involve human participants, human tissue, animals, or any procedures requiring ethical approval. Therefore, ethics approval was not required.

## 3. Results

### 3.1 SMS mesocosm experiment

Changes between T0 and T1 in individual DDX for the soil amounts in the mesocosms were found to be non-significant for both controls and treatments using mixed linear modelling, as well as PERMANOVA to test for a proportional increase in degradation productions compared to parent compounds. All mesocosms experimental data are given in S8 Table in S1 File.

#### 3.1.1 Amounts of DDX in soil.

**Fig 3:**
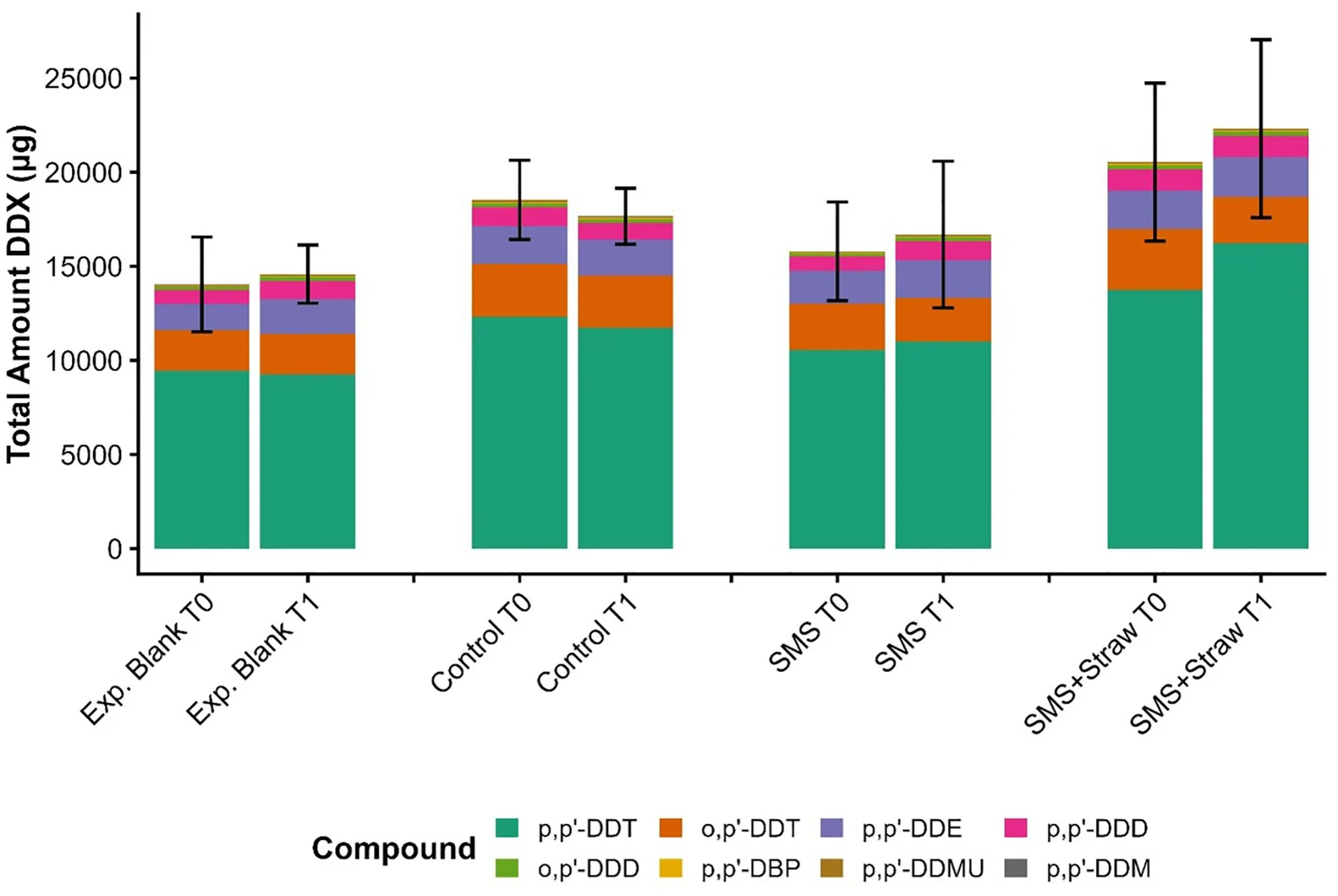
Total amount of DDX (μg) in Experimental Blank, Control, SMS treatment, and SMS+Straw treatment in the mesocosms at the beginning of the experiment (T0) and after four months of incubation (T1). Values are means with error bars showing standard deviation. n = 3 except for p,p’-DDM in Blank T0, Control T1 and SMS T0 where n = 2.

The amounts of DDX in the starting soil, the different treatments, the control and the experimental blank are visualized in Fig 3. Results are reported as amounts of DDX (μg) to avoid fluctuations due to organic matter loss (Table 2). This was possible in the mesocosms as they were closed system allowing for accurate weighing and controlling of moisture levels. No significant change was observed across treatments, and exploratory post-hoc pairwise comparisons indicated that amounts did not differ significantly between Time Point 0 and Time Point 1 for any compound in the Blank, Control, or SMS_Treatment groups (*p* > 0.05). A single significant increase in *p,p*’-DDMU was detected in the Straw+SMS treatment group (estimate = 27.13, *p* = 0.047), but this was deemed to be of low importance in the context of all the non-significant changes. The data on the individual compounds is visualized in S3 Fig.

**Table 2:** Total material mass loss per pot over the 4-month incubation period (n = 3).

Treatment	Dry weight loss (g)	Dry weight loss (%)
Experimental Blank	71 ± 150	2 ± 5
Control	400 ± 140	10 ± 4
Treatment 1	720 ± 280	17 ± 6
Treatment 2	680 ± 330	17 ± 8

Given the known heterogeneity of the substrate and the small sample volume used for analysis (1 g), the differences observed in the starting concentrations, and the variation at all time points are interpreted as random spatial variability rather than treatment effects, acknowledging the limited statistical power. Since replication was limited (*n* = 3), the results should be interpreted cautiously. Full homogenisation of the soil before sampling would have destroyed hyphal structures, limiting further growth and activity of the fungus and was therefore not done. Data, averages, and standard deviations used in analysis are shown in S8 Table in S1 File, along with theoretical T0 concentration for each treatment based on the starting soil, and the mass added in S9 Table in S1 File.

Multivariate analysis (PERMANOVA) was used to test whether the overall profile of compounds differed with Time Point, Treatment, or their interaction. The model explained 44% of the total variance in compound composition (R² = 0.437), but there was no statistically significant effect (*p* = 0.177). Per compound estimated marginal means plots are presented in S3 Fig.

#### 3.1.2 Ligninolytic enzyme activity.

The initial ligninolytic activity of the SMS before mixing into the pots was 36 ± 4 U g^-1^. Compared to activity of the spent *P. ostreatus* substrate analysed before fruiting in a preliminary test (345 ± 15 U g^-1^ (*n* = 3), the average activity was lower by a factor of 10. This reflects pronounced constraints of direct application of a mushroom production waste in a large-scale application in the field. These findings suggest that the fungi present in the *P. ostreatus* SMS was not in a particularly active state prior to inoculation. The control SMS showed no activity after autoclaving.

Ligninolytic activity was below the quantification limit for all pots at both time points (T0 and T1), with absorbance values falling below the lowest level of the calibration curve. This indicates only background enzymatic activity in all treatments, including those with SMS. However, the absence of quantitative biomass indicators such as ergosterol analysis or molecular methods (e.g., qPCR) prevents differentiation between fungal inactivity, dormancy, or competitive inhibition. Indicators of fungal viability were assessed qualitatively through visual inspection and quantitatively via ligninolytic enzyme activity. However, enzyme activity remained low indicating either insufficient reactivation of the fungal system or rapid decline in fungal viability under the laboratory conditions.

These results suggest that the oxidative enzyme systems typically attributed to white-rot fungal degradation were either not active, or present at concentrations too low to be detected under the experimental conditions. This could reflect less than ideal environmental conditions (e.g., moisture, oxygen, pH), competitive microbial interactions, or rapid decline of fungal survival post-inoculation [29]. The reactivation of spent mushroom substrate generally involves mixing with a nutrient rich, favourable substrate, (Martin et al., 2023 [30]) and the addition to soil in our experiment did not seem, from visual inspection, to facilitate the fungus’ recovery.

### 3.2 Field experiment

#### 3.2.1 Soil concentrations.

DDX data for the field experiments are given as a concentration (μg g^-1^), because amounts (μg) could not be accurately calculated in this open system with unclear boundaries. Full concentration data are shown in S10 Table in S1 File.

**Fig 4:**
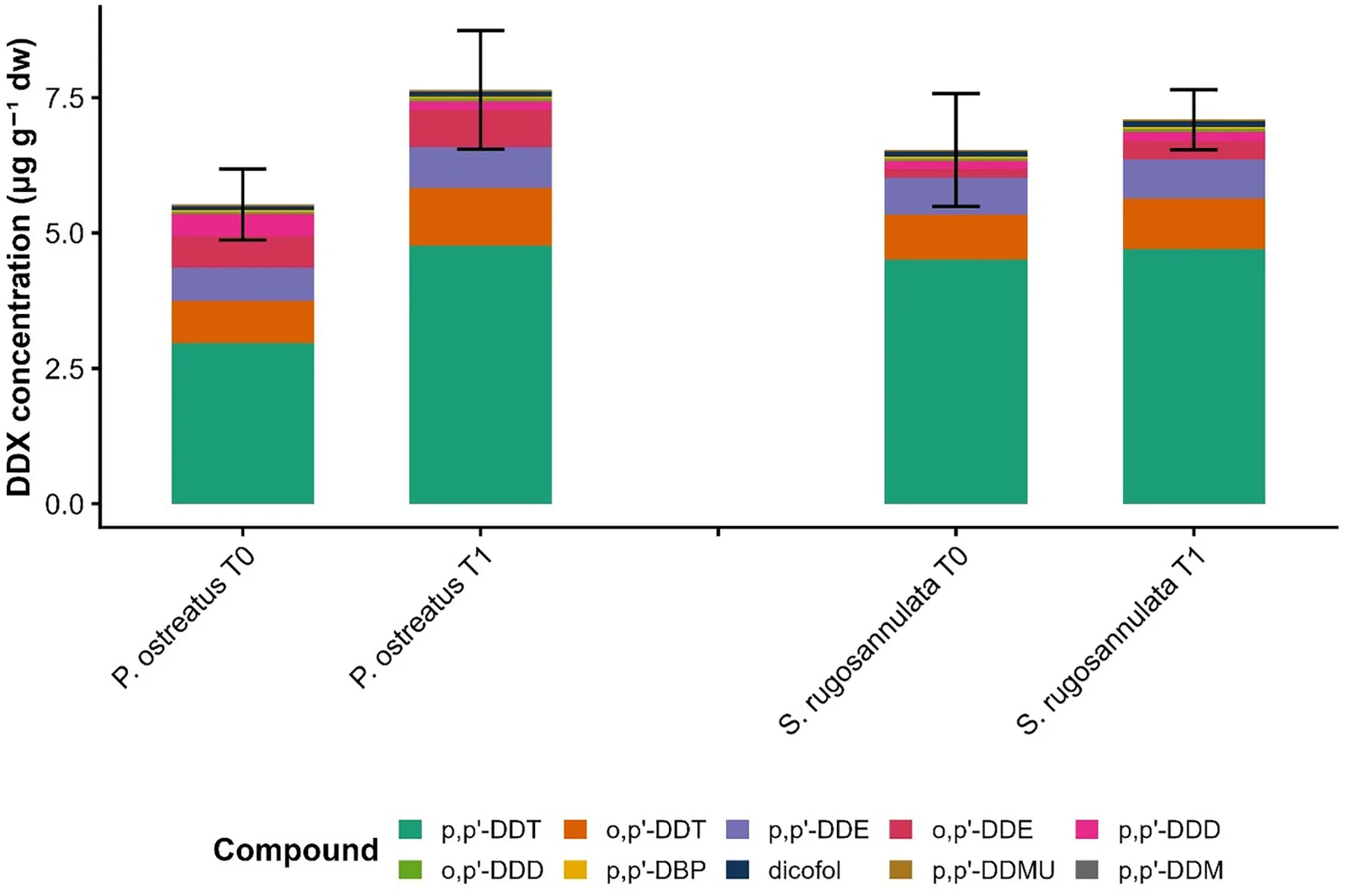
Concentration (µg g^-1^ dw) of DDX in soils of the field plots at the beginning of the experiment (T0) and after 6 weeks (T1). n = 3 except for S. rugosannulata p,p’-DDE at T0 and T1 where n = 1 and n = 2, respectively. p,p’-DDM was excluded from S. rugosannulata as levels were <LOQ.

The field trial plots of *P. ostreatus* and the *S. rugosannulata* showed no significant change in DDX concentrations between T0 and T1 (*p* > 0.05) (Fig 4). The relative standard deviation (RSD) remained low (<22%) for all plots except for *P. ostreatus* T1 where Plot 1 showed a higher DDX concentration than the others (~2 times higher) (Fig 4). This may reflect local spatial heterogeneity in the soil; however, no formal outlier tests were applied, and this remains a source of uncertainty. Otherwise, the RSD indicates representative subsampling and good replication between plots of the treatments.

#### 3.2.2 Fruiting body uptake.

**Fig 5:**
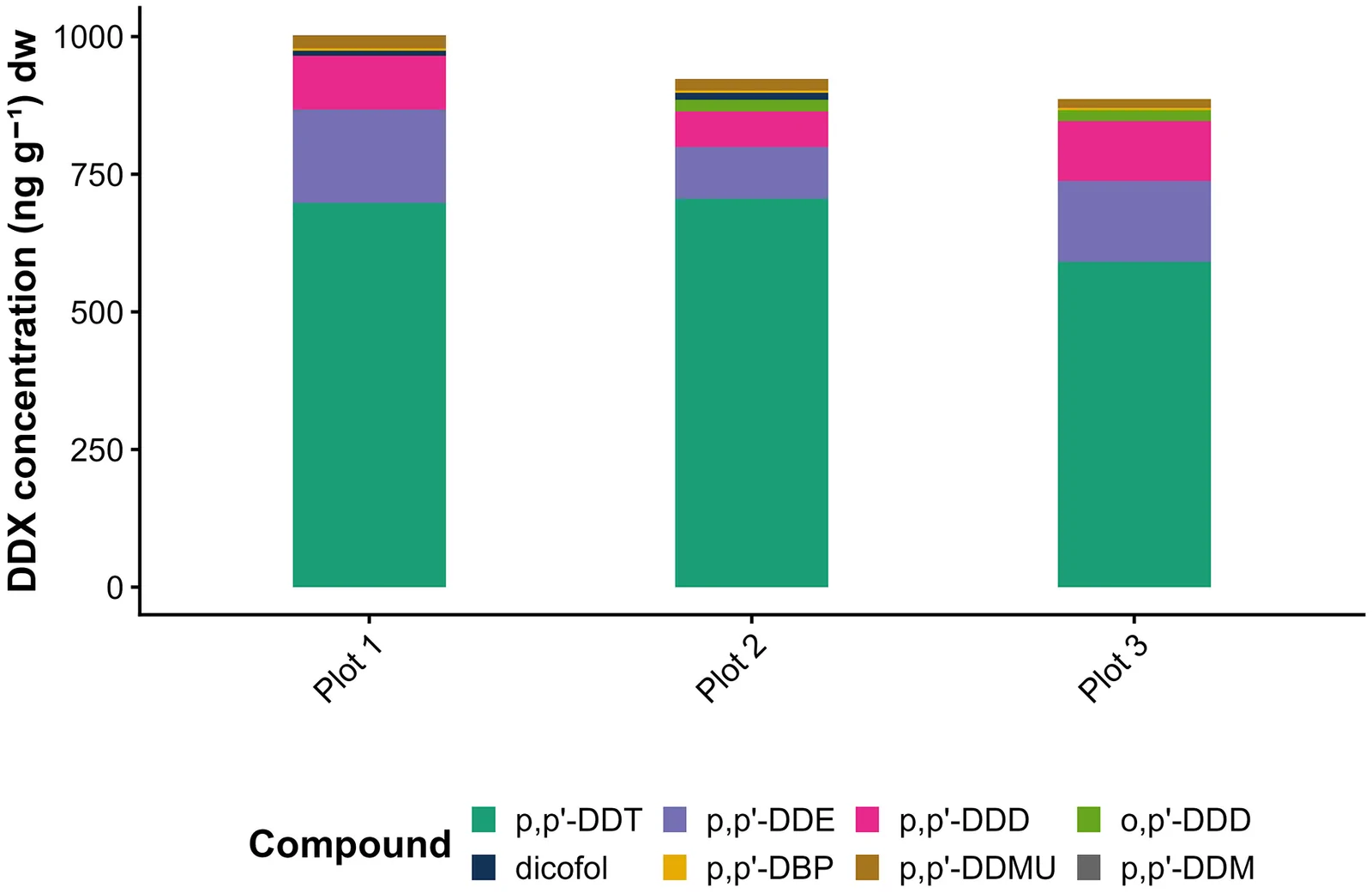
Concentration of DDX (ng g^-1^ dw) in the dried fruiting bodies of P. ostreatus, sampled from each field trial plot after 5 weeks. o,p’-DDE and o,p’-DDT were excluded due to data <LOQ and high recovery of spiked internal standards (>200%), respectively. o,p’-DDD in Plot 2 was also removed due to high recovery.

Fruiting bodies were unintentionally produced in the plots treated with *P. ostreatus*. Full data for the concentrations of DDX found in the fruiting bodies are shown in S11 Table in S1 File. One cluster of fruiting bodies was collected from each plot in week 5 and analyzed for DDX content (Fig 5). It is worth noting that the plots produced very different amounts of fruiting bodies despite the same volume of pre-inoculated substrate (20 L per plot) being spread evenly across the surface (of 2 x 2 m plot) and tilled into the upper 10–15 cm. The total amount of fruiting bodies produced in each plot were not determined as it was not within the scope of the study and to minimize disruption to the inoculated area. All DDXs analyzed were found in the fruiting bodies. The results of a PERMANOVA test show that they were taken up in proportions matching the concentrations in the surrounding soil regardless of hydrophobicity (*p* > 0.05). The concentration of DDX present within the fruiting bodies was between 5 and 10 times lower on a dry weight basis than what was present in the soil of the respective plot. Even under conservative assumptions of complete harvesting of fruiting bodies, the comparatively small fungal biomass relative to the treated soil volume (~400 L per plot) indicates that the total mass of DDX accumulated in fungal biomass would be negligible relative to the overall contaminant pool in soils.

## 4. Discussion

### 4.1 Lessons learned from SMS mesocosms – Spent mushroom substrate, quality and conditions

Previous studies have shown promising results using *P. ostreatus* SMS for the degradation of persistent organic pollutants, including DDT and related compounds [12, 19). These successes have typically been attributed to the activity of oxidative enzymes such as manganese peroxidase and laccase, which white-rot fungi use to break down complex aromatic structures while scavenging for nitrogen. However, in the mesocosm experiment, no significant enzymatic activity was detected, and no statistically significant reduction in DDX concentrations was observed under tested conditions. This is despite mimicking the mixing, dark condition, 21 °C, and ~60% moisture, which resulted in success in similar *ex-situ* in-vessel biodynamic piling experiments with PCB contaminated soils [31,32]. Although the soil was regularly mixed to maintain aeration, the use of deep pots may still have limited the development of continuous hyphal networks. A shallow, tray-based mesocosm may have better supported fungal colonization and more uniform enzyme distribution. While direct application of a waste product from mushroom production enhanced the practical relevance of the study, the lack of pre-conditioning likely contributed significantly to the low enzyme activity observed. In a study by Sadiq et al. [33], mixing SMS with soil spiked with endosulfan resulted in up to 58% degradation over the course of 35 days, and although the scale was 20 times smaller, a very similar ratio was used in this study. One of the key differences in treatment here is scale, where aeration and therefore reactivation of the SMS would have been far easier with lower volume. Even soil growing white-rot fungi generally do not thrive below approximately 15 cm depth in the soil column [34], so a mesocosm in a tray rather than a deep pot may have been better experimental design, tailored to fungal ecology.

Additionally, the same temperature and ratio of SMS to soil as Sadiq et al. [33] was used, but their soil was spiked rather than aged, and endosulfan with a log *K*_*OW*_ of 4.94 and 4.78 for α and β isomers respectively [35] is far less hydrophobic than DDX. Remediating soils contaminated with highly hydrophobic compounds like DDT presents additional challenges. Over time, these compounds sorb strongly to soil organic carbon [36,37], reducing their bioavailability and making enzymatic degradation by SMS much less likely to be effective than in freshly spiked soils [38]. However, the findings of this study should be interpreted as evidence of constraints under the tested conditions rather than as definitive evidence of treatment failure of *P. ostreatus* SMS. The present results do not allow firm separation of the contributions of low fungal activity, reduced contaminant bioavailability, and spatial heterogeneity, but they do demonstrate that these factors likely acted together to limit measurable degradation.

No specific measures were taken to enhance contaminant bioavailability because the study was intentionally designed to reflect environmentally realistic and operationally simple field-relevant conditions suitable for large-scale application. The aim was to evaluate whether fungal substrates could perform under field-relevant conditions without additional amendments, intensive soil manipulation or SMS pre-conditioning. As a result, surfactant addition was not included, even though it may have increased desorption of aged DDT and improved fungal access to the contaminant. This design choice increases the practical relevance of the study, but it also means that limited bioavailability likely contributed to no degradation observed.

One potential explanation for the limited effectiveness observed here is the biological and physical condition of the SMS at the time of inoculation. While SMS is often promoted as a low-cost, abundant remediation agent, not all SMS is equally suitable without pre-conditioning. To mimic the field application setting in the laboratory, the SMS obtained from mushroom production was used directly in this experiment. It was wet, compacted, and relatively inactive, lacking the fluffy white hyphal growth typical of pre-conditioning fungal substrate. Moreover, physical homogenisation of the substrate, in which visible hyphal structures were diluted within the bulk material during mixing could significantly reduce fungal viability and enzyme expression. It is worth noting that the remaining spent mushroom substrate developed extensive fluffy white hyphal growth during its long-term cold storage (+7°C), in keeping with the cyclical nature of the fungal lifecycle. Typically, 2–3 flushes of mushrooms are produced from a well-functioning substrate, and the highest enzyme activity is observed before fruiting, in the process of colonizing a fresh substrate (Bellettini et al., 2019 [29]). Had the application of the spent mushroom substrate been postponed until renewed hyphal growth was more firmly established, it is probable that the mesocosm experiment would have yielded different results, potentially enhancing fungal activity and promoting degradation processes.

For SMS to become metabolically active again in soil, it typically requires aeration and fresh organic input of organic matter, such as straw, woodchips or even coffee grains, to stimulate hyphal regrowth and enzyme production. In this study, only one treatment (T2) received an additional carbon source (wet straw), and even there, activity remained undetectable, possibly because the SMS had already deteriorated. Straw was used due to pre-trials indicating its superiority to woodchip in promoting ligninolytic enzyme production in the short term, but other substrates such as sawdust or woodchip could have also been explored. It is also possible that because supplementation was done simultaneously with inoculation in soil, the competitive microbial communities in the soil colonised the substrate first and inhibited fungal regrowth. Thus, another approach would have been to mix in straw and only inoculate the soil once the enzyme activity had increased, and hyphal growth could be observed. This approach was therefore used in the field trial.

This study highlights the material quality as an important limitation for SMS-based remediation. SMS that may be perfectly suitable as a soil amendment for nutrient supply or organic matter enrichment may not be appropriate for remediation if the colonising fungus is no longer viable or the ligninolytic enzymes have degraded. These typically have a half-life of less than a week, so if the fungus is not producing more, these will not be replenished [11]. Future studies should include quantitative biomass indicators such as ergosterol or molecular tools to better resolve fungal viability and activity under soil conditions and consider pre-treatment steps like reactivation through aeration and supplementation to optimise conditions for fungal activity.

No degradation observed in this study may reflect both limited hyphal development and the contrast between aged field contamination and the freshly spiked soils used in the successful laboratory study by Purnomo et al. [12]. In freshly spiked soils, contaminants are generally more bioavailable, whereas aging promotes strong sorption and diffusion into soil organic matter, thereby reducing desorption and bioavailability. This behavior is well documented for hydrophobic organic contaminants, such as DDT, and often results in lower degradation efficiency in field soils than in spiked soils [39].

### 4.2 Lessons learned from field experiment – Field techniques, and challenges with upscaling

The second tested remediation technique included tilling of inoculated mushroom substrate into the upper DDT contaminated soil in a 6-week field trial field trial to assess whether fungal establishment could be initiated under environmentally realistic conditions within a short monitoring period during summer time. A target spawn-to-soil ratio of ~5% (v/v), i.e., a substrate addition of 20% (5% spawn and 15% field colonized straw), was selected as in previous success studies for other organic contaminants [31,32,40]. However, larger application rates may have influenced the colonization outcome. Under tested conditions, no measurable degradation of DDT in the soil by *P. ostreatus* and *S. rugosoannulata* was detected, which likely stems from several ecological and environmental constraints.

Firstly, *P. ostreatus* is a wood-decomposing fungus poorly adapted to mineral soil environments. Particularly in compacted agricultural soils with little aeration, *P. ostreatus* was at a disadvantage. In field conditions, it faces competition from established native soil microbiota and must operate under unfamiliar and often suboptimal conditions of moisture, temperature, and nutrient availability. Unlike in controlled *in vitro* environments [41], the fungus struggles to permeate the soil matrix, leading to poor colonization and patchy distribution.

Another limitation may have been the quantity of spawn and substrate applied. Moreover, straw, used as a substrate in the field trial, might not support sustained ligninolytic enzyme production and fungus survival as effectively as woodchips may have. Although the inoculation ratio used was similar to that in a successful *ex-situ* in-vessel biodynamic piling PAH remediation trial by Di Gregorio et al. [32], this proportion may be insufficient in *in-situ* field settings where fluctuating temperatures, variable rainfall, and ongoing biotic disturbance from soil fauna and vegetation can interfere [38]. *S. rugosoannulata*, while better adapted to soil habitats, is a slower colonizer and appeared unable to establish aggressively enough to have an impact.

Visual assessments of soil cores support these observations. In *P. ostreatus* plots, mycelial growth was generally confined to the top 2–3 cm, whereas in *S. rugosoannulata* plots, vertical growth reached up to 7 cm after six weeks. However, in both cases, visual colonization was mostly restricted to patches of substrate, with minimal spread into the surrounding soil. Given this limited colonization, the quantity and distribution of extracellular enzymes were likely too sparse to meaningfully affect DDX concentrations in soil. Given that DDX contamination is distributed throughout the upper soil layer (0–20 cm), shallow colonization restricts contact between fungal enzymes and target compounds, thereby limiting degradation potential deeper in the soil. Moreover, even though enzyme activity likely did occur in the soil, the enzymes themselves are short-lived in soil environments, subject to denaturation or microbial metabolization within days of release [42]. Future studies should include quantitative biomass indicators such as ergosterol or molecular tools to better monitor fungal viability and activity under soil conditions.

Competition with native soil microbiota is another important limiting factor. Indigenous microbial communities are well adapted to local soil conditions and can rapidly colonize available substrates, potentially outcompeting introduced fungi for nutrients and space. This competition could suppress fungal establishment and reduce enzyme production, particularly because the introduced substrates were not pre-conditioned and fungal biomass was relatively low. Environmental variability, fungal colonization depth and competition with native microbiota in soils likely created significant constraints on fungal establishment and activity in field. Interestingly, signs of fruiting body formation were observed in *P. ostreatus* plots. At week 4, one replicate began forming fruiting bodies, and by week 5, following a period of heavy rainfall and cooler temperatures, all three *P. ostreatus* plots exhibited fruiting (Fig 2). This indicates that the inoculated straw remained viable and metabolically active. However, it also suggests that the fungi were allocating energy to reproduction over soil exploration or scavenging for nutrients. While accumulation of DDX was observed in fruiting bodies, its magnitude is too small to be considered a remediation technique. In contrast, *S. rugosoannulata* did not fruit during the study period. This species typically fruits between spring and autumn, and inoculation in late autumn in our case therefore might have missed its productive window. The content of DDT in the mushrooms, while being up to 10 times lower than the surrounding soil, still exceed safe limits for food, which is another important consideration when attempting mycoremediation without restricting access to the remediation site.

### 4.3 Valuable insights for future implementing fungal-based remediation under natural conditions

In successful lab- and pilot-scale experiments, soils were commonly mixed with 10% or 20% spent substrate of *P. ostreatus* by weight [31,32,40]. In a pilot-scale field trial using 10 tonnes of historically contaminated soil, Siracusa et al. [31] reported 94% PCB degradation after 8 months of incubation. Another successful field trial by Di Gregorio et al. [32], involving 7 tonnes of historically contaminated soil incubated for 1, 2, and 4 months, achieved 51%, 72%, and 98% degradation of PAHs, respectively. In contrast, Purnomo et al. [12] used a 1:1 dry weight ratio, achieving 48% degradation of DDT after 28 days of incubation in a lab-scale experiment using 2 g of artificially contaminated soil, highlighting the wide range of ratios that could potentially be applied in practice at large scales. However, no optimal soil-to-substrate ratio has been identified in the literature for effective biodegradation of persistent organic pollutants with help of spent substrate of *P. ostreatus*. The ratio likely influences the bioremediation potential of the substrate. In our experiments, 20% colonized substrate was tested in the pot trial and a substrate addition of 20% (5% spawn and 15% field colonized straw) in the field trial, but both yielded no degradation under tested conditions.

While this study did not observe degradation of DDX using SMS alone, there is still potential for SMS to serve as a complementary amendment in remediation strategies for persistent organic pollutants. If properly conditioned, through optimal storage, aeration, physical loosening, and the addition of fresh carbon sources, SMS could help re-establish fungal activity and provide both enzymatic input and organic matter to support soil treatment. This approach could also be more cost-effective than using fresh pre-fruiting spawn and substrate mixture, though both options could be further tested as amendments in field-scale remediation trials.

One potential remediation strategy is the integration of SMS into *ex-situ* biopiling systems, a widely used technique for remediating hydrocarbon-contaminated soils through aerobic microbial degradation in the field [32]. SMS could improve aeration, moisture retention, and heat generation, which can stimulate microbial activity and potentially support fungal regrowth. Its fibrous structure and residual nutrients may further improve soil pore structure and oxygen flow, both critical for microbial and fungal degradation processes. However, DDT-contaminated sites often cover large areas, so socio-economic considerations are important. Implementing such remediation at a large scale may require substantial labor and fossil fuel input, potentially increasing air emissions. Additionally, some transformation products of DDT are toxic, and treatment could increase mobility of the compounds, underscoring the need for careful risk assessment. Complete degradation is essential, and the potential for leaching should be evaluated under controlled laboratory conditions before field application.

Another possible avenue is combining SMS with iron-based amendments, such as zero-valent iron, which are frequently used in treatments targeting chlorinated organic pollutants like DDT [43]. While these techniques typically rely on redox manipulation, they could be enhanced by the organic matter and microbial communities hosted by SMS. Additionally, the physiological state of the fungi, i.e., enzyme production, should be followed and SMS used when it is in its optimal state.

Although *P. ostreatus* and similar species, have shown the ability to degrade DDX under lab conditions [12,41]), applying them at field scale might require a different strategy. Simply introducing a new species into a field environment may not be effective, due to the challenges of establishment in open soils. A two-step process could be explored, where contaminated soil is excavated and treated under controlled conditions, with stable moisture, temperature, and no disturbance by soil flora or fauna [44]. However, employing such *ex-situ* techniques can prove to be exceedingly costly, both for society and the stakeholders involved, owing to the vast quantities of DDT-contaminated soil present at these sites. Alternatively, a soil washing step could be used, such as with a biosurfactant, to mobilize the DDX to a liquid phase, and this extract could then be treated in liquid with the fungi [2]. While this approach can increase bioavailability for fungal degradation, it also introduces the potential risk of spreading DDX to unintended areas, so careful monitoring of leachability and containment measures would be essential. *P. ostreatus* has been successfully applied in a trickle-bed reactor to treat PCB-contaminated water, achieving up to 80% degradation [45]. While trickle-bed reactors may not be suitable for treating contaminated soils directly, they could potentially be used to process soil-wash effluents where DDX have been extracted using surfactants. This approach would allow fungal bioreactors to be leveraged in a controlled environment, reducing the limitations imposed by in-soil competition and environmental variability. Laboratory-scale tests using bioavailable DDX should be conducted first, measuring enzyme production and respiration rates before scaling up. Additionally, testing conditions for complete degradation (mineralization to CO_2_) under controlled laboratory settings can help optimize treatment parameters, such as substrate ratios, moisture, and aeration, providing a foundation for safer and more effective field applications.

Such hybrid approaches are promising avenues in the remediation field and may provide a way to improve treatment efficiency while reducing chemical input. Rather than viewing mycoremediation as a single-solution treatment, it may be best applied as part of a combined treatment system for soil remediation, especially in the context of sustainable, low-waste, and circular economy models.

## 5. Conclusions

The results of the field trial underscore the complexities of scaling up fungal-based remediation strategies from controlled, small scale lab studies to open, dynamic soil systems. Factors such as environmental variability, competition with native microbiota, fungal ecology, inoculum viability, substrate compatibility, fungal colonization depth, and site-specific disturbances must all be considered. Under the conditions tested, no measurable degradation of DDT or its transformation products was observed in either the pot experiment or the field trial. However, these findings are specific to the conditions tested in this study and should not be taken as evidence that mycoremediation is ineffective for aged DDT-contaminated soils under other conditions.

Successful bioremediation of such sites likely requires additional measures to enhance fungal establishment and maintain enzyme activity. In addition, the bioavailability of DDX in historically contaminated, aged soils should be considered, as limited availability may constrain degradation and may need to be addressed through strategies such as surfactant addition or soil mixing. Furthermore, pre-fruiting spawn may not be able to establish well enough in open field conditions for remediation purposes but may well be able to treat soil within in-vessel systems. This study highlights the need for establishing thoroughly controlled systems in bioremediation treatment. Care needs to be taken to ensure consistent quality of the used biological material potentially impacting cost and labour of the chosen bioremediation strategy. While no significant degradation of DDT or its transformation products was observed in this study, the findings provide insight into the limitations of current methods and highlight the need for well-controlled and possibly hybrid remediation systems. Careful consideration of biological material quality and system design will be essential to making fungal bioremediation of DDT contaminated soils both effective and scalable.

## Supporting information

S1 File This file contains soil characterization data, supporting figures, chemical analysis details, recovery data, DDX concentration/amount tables, and statistical output supporting the manuscript. (DOCX)

S1 Fig Pot-scale mesocosm experiment photos. (PNG)

S2 Fig Field trial site photos. (PNG)

S3 Fig Estimated marginal means plots for individual DDX compound amounts before and after SMS mesocosm incubation. (PNG)
