# Supplementary material for: Lessons learned from two applied approaches for treatment of aged DDT-contaminated soil with white-rot fungi
Source: PLoS One. 2026 Sep 11;21(9):e0357404. doi: 10.1371/journal.pone.0357404 (PMC13567788; doi:10.1371/journal.pone.0357404)
Supplement: Supplementary file 1 [file pone.0357404.s001.docx]

**Supplementary Information**

**Evaluation of two applied approaches for treatment of aged DDT-contaminated soil with white-rot fungi**

S. Casey^a*^, K. Wiberg^a^, A-K. Dahlberg^a,b^ , M. Hultberg^c^, A. Enell^d^, Y. Volchko^e^

^a^Department of Aquatic Sciences and Assessment, Swedish University of Agricultural Sciences (SLU), SE-750 07 Uppsala, Sweden

^b^IVL Swedish Environmental Research Institute, P.O. Box 210 60, SE-100 31 Stockholm, Sweden

^c^Department of Biosystems and Technology, Swedish University of Agricultural Sciences SE-234 56 Alnarp, Sweden

^d^Swedish Geotechnical Institute (SGI), SE-581 93 Linköping, Sweden.

^e^Division of Geology and Geotechnics, Department of Architecture and Civil Engineering, Chalmers University of Technology, SE-412 95 Gothenburg, Sweden

*Corresponding author: stephanie.casey@slu.se

**Contents**

Methods description for basic soil characterisation

Table S1: Soil characterization data for the Kolleberga forest nursery.

Figure S1: Pot-scale mesocosm experiment photos.

Figure S2: Field trial site photos.

Table S2: Details for chemicals and materials used for chemical analysis and enzyme assays

Table S3: Target compounds, ^13^C-labelled internal standards (IS) (final concentration 1 ng μL^-1^), and ^13^C-labelled recovery standard (RS), along with vendors, purities, and precursor and product ions

Table S4: Quality control test of the degree of DDT degradation into DDD in the GC-injector using a *p,p’*-DDT solution and checking for the formation of *p,p’*-DDD

Table S5: DDX recoveries in soil analysis in mesocosm and field study

Table S6: DDX recoveries (%) in soil field study experiment soil evaluation.

Table S7: DDX recoveries (%) in the field experiment fruiting body evaluation

Table S8: Soil amounts of DDX before (T0) and after (T1) treatment in the mesocosm study

Figure S3: Estimated marginal means plot presenting individual DDX compound amounts (μg) before and after SMS mesocosm incubation

Table S9: Theoretical soil amounts of DDX in before (T0) treatment in the mesocosm study, compared to experimental soil amounts

Table S10: Soil amounts of DDX before (T0) and after (T1) treatment in the field scale study.

Table S11: The concentration of DDX within the fruiting bodies of *P. ostreatus*

**Methods description for basic soil characterisation**

pH was determined in a 1g wet weight (ww) soil to 10 mL deionized water slurry using a pH meter (S20 SevenEasy pH, Mettler Toledo AB, Stockholm, Sweden). Organic carbon content was measured using loss on ignition (LOI); combustion soil at 505 °C for 4 h. Soil texture was assessed by filtration, and total nitrogen and total carbon were determined by dry combustion (1350 °C) and elemental analysis using a TruMac instrument (LECO Corporation, MI, USA).

**Table S1**: Soil characterization data for the Kolleberga forest nursery.

| **pH** |  | **Org-C** | **Clay** | **Silt** | **Sand** | **N-tot** | **C-tot** | **Water content** |
| --- | --- | --- | --- | --- | --- | --- | --- | --- |
|  |  | % | % | % | % | (g kg^-1^ dw) | (g kg^-1^ dw) | % |
| 6.3 ± 0.3 |  | 3.1 ± 0.12 | 3 | 16 | 82 | 0.85 ± 0.014 | 15 ± 0.12 | 15±1.5 |

Org-C: organic carbon, N-tot: total nitrogen, C-tot: total carbon

**Figure S1**: Pot-scale experiment photos. A) Pot mesocosms setup: soil, SMS and substrate mix covered with mesocosm fabric; B) Mixed soil, spent mushroom substrate (SMS) and substrate; C) SMS layer covering soil layer (pre-mixing).

**A**

**B**

**C**

**Figure S2**: Field trial site photos. A) *S. rugosannulata* spawn and colonised straw after 1 week inoculation; B) *P. ostreatus* spawn and colonised straw after 1 week inoculation; C) Inoculated substrate spread over plot, pre-mixing; D) Pre- and post-mixing plots of *P. ostreatus*, with hand-tiller; E) Plot covered with barrier fabric; F) *S. rugosannulata* plot a post sampling at 4-months (T1).

**C**

**A**

**B**

**D**

**Table S2**: Details for chemicals and materials used for chemical analysis and enzyme assays

| **Product** | **Chemical name** | **Vendor** | **Product number** | **CAS number** |
| --- | --- | --- | --- | --- |
| Calcium carbonate | CaCO_3_ | Sigma-Aldrich | C4830 | 471-34-1 |
| Whatman® cellulose extraction thimbles | - | Merck | WHA10350243 | - |
| Dichloromethane | CH_2_Cl_2_ | VWR International | 1.06054.2500 | 75-09-2 |
| *n*-Hexane | CH_3_(CH_2_)_4_CH_3_ | VWR International | 1.04371.2500 | 110-54-3 |
| Activated granular copper | Cu | Merck | 311405 | 7440-50-8 |
| Aluminum oxide | Al_2_O_3_ | Merck | 544833 | 1344-28-1 |
| Silicon dioxide | SiO_2_ | Merck | 922587 | 7631-86-9 |
| Sodium sulphate | Na_2_SO_4_ | Merck | 238597 | 7757-82-6 |
| Isooctane | (CH_3_)_2_CHCH_2_C(CH_3_)_3_ | Merck | 1.15440 | 540-84-1 |
| 3-Methyl-2-benzothiazolinone-hydrazonehydrochloride (MBTH) | C_8_H_10_ClN_3_S | Merck | 1.04527 | 4338-98-1 |
| 4-(Dimethylamino)-benzaldehyde (DMAB) | C_20_H_27_NO_3_ | Merck | D2004 | 172611-73-3 |
| Manganese-dependant lignin peroxidase (MnP) | - | Merck | 803057 | 114995-15-2 |
| Ethylenediaminetetraacetic acid (EDTA) | (HO_2_CCH_2_)_2_NCH_2_CH_2_N(CH_2_CO_2_H)_2_ | Merck | E6758 | 60-00-4 |
| Hydrogen peroxide solution (30%) | H_2_O_2_ | Merck | 31642-M | 7722-84-1 |
| Sodium acetate | C_2_H_3_NaO_2_ | Merck | 241245 | 127-09-3 |
| Sodium succinate dibasic hexahydrate | NaOOCCH_2_CH_2_COONa · 6H_2_O | Merck | S2378 | 6106-21-4 |
| Sodium DL lactate solution | CH_3_CH(OH)COONa | Merck | L4263 | 72-17-3 |

**Table S3**: Target compounds, ^13^C-labelled internal standards (IS) (final concentration 1 ng μL^-1^), and ^13^C-labelled recovery standard (RS), along with vendors, purities, and precursor and product ions

| **Compounds** | **Vendor** | **Chemical purity (%)** | **Precursor ion** | **Product ion** | **Internal standard (IS)** |
| --- | --- | --- | --- | --- | --- |
| **Target compounds** |  |  |  |  |  |
| *o,p′-*DDT | Cambridge Isotope Laboratories, Inc., Massachusetts, USA. | 99.2 | 235 | 165 | ^13^C-*o,p*′*-*DDT |
| *p,p′-*DDT |  | 99.9 | 235 | 165 | ^13^C-*p,p*′*-*DDT |
| *o,p′-*DDD |  | 97.5 | 235 | 165 | ^13^C-*o,p*′*-*DDD |
| *p,p′-*DDD |  | 99.2 | 235 | 165 | ^13^C-*p,p*′*-*DDD |
| *o,p′-*DDE |  | 99.7 | 246 | 176 | ^13^C-*o,p*′*-*DDE |
| *p,p′-*DDE |  | 99.5 | 246 | 176 | ^13^C-*p,p*′*-*DDE |
| *p,p′-*DDMU | Sigma-Aldrich | 98.9 | 212 | 176 | ^13^C-*o,p*′*-*DDE |
| *p,p′-*DDM | LGC Standards, Guilford, UK | 99.5 | 201 | 165 | ^13^C-*o,p*′*-*DDE |
| *p,p′-*DBP | Sigma-Aldrich | 98.7 | 139 | 111 | ^13^C-*o,p*′*-*DDE |
| Dicofol | Cambridge Isotope Laboratories | 99.48 | 251 | 139 | ^13^C*-*Dicofol |
|  |  |  |  |  |  |
| **Internal standards (IS)** |  |  |  |  |  |
| 7 DDX solution |  |  |  |  |  |
| ^13^C-*o,p′-*DDT | Cambridge Isotope Laboratories, Inc., Massachusetts, USA. | 99.5 | 247 | 177 |  |
| ^13^C-*p,p′-*DDT |  | >98 | 247 | 177 |  |
| ^13^C-*o,p′-*DDD |  | 96.2 | 247 | 177 |  |
| ^13^C-*p,p′-*DDD |  | 98.2 | 247 | 177 |  |
| ^13^C-*o,p′*-DDE |  | 99.6 | 258 | 188 |  |
| ^13^C-*p,p′-*DDE |  | >98 | 258 | 188 |  |
| ^13^C-Dicofol |  | 99.5 | 263 | 145 |  |
|  |  |  |  |  |  |
| **Recovery standard (RS)** |  |  |  |  |  |
| ^13^C-PCB 97 | Cambridge Isotope Laboratories, Inc., Guelp, Onatorio, Canada. | 99% | 340 | 268 |  |
| ^13^C-PCB 188 |  | 99% | 406 | 336 |  |

**Table S4**: Quality control test of the degree of DDT degradation into DDD in the GC-injector using a *p,p’*-DDT solution and checking for the formation of *p,p’*-DDD

| GC-MS/MS run | *p,p’*-DDT pre-run  (ng L^-1^) | *p,p’*-DDD pre-run  (ng L^-1^) | **Degradation pre-run**  **(%)** | *p,p’*-DDT post-run  (ng L^-1^) | *p,p’*-DDT post-run  (ng L^-1^) | **Degradation post-run**  **(%)** |
| --- | --- | --- | --- | --- | --- | --- |
| SMS | 240 | 15 | **6.4** | 280 | 12 | **4.5** |
| Field Trial | 240 | 15 | **6.4** | 210 | 20 | **9.9** |
| Mushroom | 960 | 61 | **6.4** | 1200 | 91 | **7.9** |

Pre-run: After the calibration curve but before the samples; Post-run: after all samples of that batch

**Table S5**: DDX recoveries (%) in soil in mesocosm experiments. Values outside the recovery limits (<50% or >200%) were excluded from further evaluation. *indicates a removed value, and where *n*=2 in averages and standard deviation. Dicofol and *o,p*’-DDE were excluded due to all values violating the recovery limits.

| **Mesocosm soil** r**ecovery (%)** | *p,p'*-DDT | *o,p'*-DDT | *p,p'*-DDD | *o,p'*-DDD | *p,p'*-DDE |
| --- | --- | --- | --- | --- | --- |
| Starting Soil-a | 166 | 75 | 92 | 69 | 97 |
| Starting Soil-b | 114 | 58 | 87 | 67 | 94 |
| Starting Soil-c | 111 | 57 | 89 | 70 | 92 |
| Exp. blank-a T0 | 186 | 114 | 181 | 146 | 193 |
| Exp. blank-b T0 | 136 | 71 | 96 | 79 | 103 |
| Exp. blank-c T0 | 113 | 61 | 94 | 74 | 97 |
| Exp. blank-a T1 | 124 | 65 | 93 | 75 | 94 |
| Exp. blank-b T1 | 114 | 70 | 115 | 90 | 118 |
| Exp. blank-c T1 | 118 | 65 | 93 | 73 | 94 |
| Control-a T0 | 139 | 77 | 106 | 87 | 111 |
| Control-b T0 | 21* | 25* | 64 | 41* | 63 |
| Control-c T0 | 123 | 66 | 96 | 73 | 100 |
| Control-a T1 | 144 | 72 | 94 | 79 | 102 |
| Control-b T1 | 133 | 72 | 98 | 76 | 103 |
| Control-c T1 | 132 | 69 | 94 | 70 | 96 |
| SMS Treatment-a T0 | 104 | 59 | 93 | 69 | 96 |
| SMS Treatment-b T0 | 128 | 66 | 94 | 71 | 100 |
| SMS Treatment-c T0 | 151 | 73 | 95 | 72 | 97 |
| SMS Treatment-a T1 | 144 | 72 | 94 | 77 | 100 |
| SMS Treatment-b T1 | 137 | 89 | 145 | 119 | 148 |
| SMS Treatment-c T1 | 142 | 73 | 99 | 84 | 107 |
| SMS+Straw Treatment-a T0 | 129 | 73 | 97 | 96 | 101 |
| SMS+Straw Treatment-b T0 | 140 | 80 | 100 | 91 | 104 |
| SMS+Straw Treatment-c T0 | 126 | 71 | 101 | 92 | 103 |
| SMS+Straw Treatment-a T1 | 129 | 70 | 96 | 83 | 101 |
| SMS+Straw Treatment-b T1 | 90 | 58 | 92 | 77 | 93 |
| SMS+Straw Treatment-c T1 | 134 | 72 | 99 | 87 | 103 |
| **Average** | 131 | 71 | 100 | 83 | 104 |
| **Stdev** | 19 | 11 | 20 | 17 | 22 |
| **Min** | 90 | 57 | 64 | 67 | 63 |
| **Max** | 186 | 114 | 181 | 146 | 193 |

**Table S6**: DDX recoveries (%) in the field study experiment soil evaluation. Values outside the recovery limits (<50% or >200%) were excluded from further evaluation.

| **Field trial soil recovery (%)** | *o,p'*-DDT | *p,p*-DDT | *o,p'*-DDE | *p,p'*-DDE | *o,p'*-DDD | *p,p'*-DDD | Dicofol |
| --- | --- | --- | --- | --- | --- | --- | --- |
| *P. ostreatus* plot 1 T0 | 123 | 144 | 119 | 117 | 88 | 82 | 79 |
| *P. ostreatus* plot 2 T0 | 105 | 112 | 100 | 97 | 86 | 84 | 78 |
| *P. ostreatus* plot 3 T0 | 100 | 101 | 99 | 97 | 88 | 88 | 74 |
| *P. ostreatus* plot 1 T1 | 81 | 101 | 94 | 91 | 84 | 83 | 86 |
| *P. ostreatus* plot 2 T1 | 94 | 97 | 114 | 109 | 105 | 108 | 129 |
| *P. ostreatus* plot 3 T1 | 127 | 130 | 97 | 100 | 90 | 78 | 74 |
| *S. rugosannulata* plot 1 T0 | 120 | 103 | 79 | 72 | 75 | 82 | 120 |
| *S. rugosannulata* plot 2 T0 | 169 | 143 | 103 | 86 | 98 | 108 | 137 |
| *S. rugosannulata* plot 3 T0 | 148 | 134 | 97 | 89 | 99 | 111 | 156 |
| *S. rugosannulata* plot 1 T1 | 150 | 132 | 93 | 82 | 96 | 111 | 162 |
| *S. rugosannulata* plot 2 T1 | 150 | 133 | 90 | 82 | 92 | 105 | 150 |
| *S. rugosannulata* plot 3 T1 | 165 | 140 | 96 | 86 | 98 | 115 | 129 |
| Average | 128 | 122 | 98 | 92 | 92 | 96 | 114 |
| Stdev | 28 | 18 | 10 | 12 | 8 | 14 | 33 |
| Min | 81 | 97 | 79 | 72 | 75 | 78 | 74 |
| Max | 169 | 144 | 119 | 117 | 105 | 115 | 162 |

**Table S7**: DDX recoveries (%) in the field soil experiment fruiting body evaluation. Values outside the recovery limits (<50% or >200%) were excluded from further evaluation. *indicates a removed value, and where *n*=2 in averages and standard deviation.

| **Field trial fruiting body recovery (%)** | *o,p'*-DDT | *p,p-*DDT | *o,p'*-DDE | *p,p'*-DDE | *o,p'*-DDD | *p,p'*-DDD | Dicofol |
| --- | --- | --- | --- | --- | --- | --- | --- |
| Plot 1 | 200* | 147 | 150 | 122 | 177 | 76 | 146 |
| Plot 2 | 226* | 149 | 120 | 139 | 251* | 61 | 148 |
| Plot 3 | 200* | 128 | 149 | 113 | 150 | 79 | 42* |
| **Average** | 209 | 141 | 140 | 125 | 193 | 72 | 112 |
| **Stdev** | 12 | 9 | 14 | 11 | 42 | 8 | 49 |
| **Min** | 200 | 128 | 120 | 113 | 150 | 61 | 42 |
| **Max** | 226 | 149 | 150 | 139 | 251 | 79 | 148 |

**Table S8**: Soil amounts (μg) of DDT and its transformation products before (T0) and after (T1) treatment in the mesocosm study. Values outside of recovery limits (<50% or >200%) were excluded from data evaluation (not included in calculations of averages and standard deviation). Dicofol and *o,p'*-DDE data are missing due to too low recovery values.

| **Soil DDX amount (μg)** | *p,p'*-DDT | *o,p'*-DDT | *p,p'*-DDD | *o,p'*-DDD | *p,p'*-DDE | *p,p'*-DDMU | *p,p'*-DDM | *p,p*'-DBP |
| --- | --- | --- | --- | --- | --- | --- | --- | --- |
| Starting Soil-a | 14000 | 3800 | 1200 | 270 | 2700 | 110 | 13 | 74 |
| Starting Soil-b | 11000 | 2800 | 940 | 200 | 2000 | 100 | 11 | 55 |
| Starting Soil-c | 12000 | 3000 | 1300 | 310 | 2100 | 130 | 17 | 62 |
| Control-a T0 | 12000 | 1400 | 310 | 110 | 2400 | 150 | 12 | 54 |
| Control-b T0 | <RL | <RL | <RL | <RL | <RL | <RL | <RL | <RL |
| Control-c T0 | 11000 | 2500 | 850 | 190 | 1800 | 110 | 11 | 50 |
| Control-a T1 | 13000 | 3300 | 1100 | 240 | 2200 | 120 | 13 | 68 |
| Control-b T1 | 11000 | 2400 | 850 | 190 | 1800 | 89 | 10 | 51 |
| Control-c T1 | 11000 | 2600 | 760 | 160 | 1800 | 67 | <LOQ | 42 |
| Exp. blank-a T0 | 7400 | 1500 | 520 | 120 | 1100 | 66 | 5.7 | 24 |
| Exp. blank-b T0 | 12000 | 2700 | 940 | 220 | 1800 | 100 | 12 | 65 |
| Exp. blank-c T0 | 9000 | 2100 | 690 | 160 | 1400 | 75 | <LOQ | 41 |
| Exp. blank-a T1 | 11000 | 2600 | 1100 | 270 | 2100 | 95 | 10 | 52 |
| Exp. blank-b T1 | 8400 | 1900 | 980 | 230 | 1600 | 120 | 9.6 | 23 |
| Exp. blank-c T1 | 8400 | 2000 | 790 | 200 | 1700 | 69 | 8.6 | 41 |
| SMS_Treatment-a T0 | 7700 | 1700 | 640 | 140 | 1300 | 67 | 8.8 | 26 |
| SMS_Treatment-b T0 | 12000 | 2800 | 1000 | 210 | 2000 | 120 | 9.3 | 44 |
| SMS_Treatment-c T0 | 12200 | 2800 | 610 | 140 | 2000 | 59 | <LOQ | 42 |
| SMS_Treatment-a T1 | 14000 | 2800 | 1100 | 250 | 2500 | 92 | 9.7 | 56 |
| SMS_Treatment-b T1 | 6700 | 1300 | 620 | 140 | 1200 | 54 | 4.8 | 17 |
| SMS_Treatment-c T1 | 13000 | 2800 | 1300 | 270 | 2500 | 130 | 10 | 49 |
| SMS+Straw Treatment-a T0 | 9100 | 3500 | 1200 | 230 | 2100 | 95 | 11 | 86 |
| SMS+Straw Treatment-b T0 | 17000 | 3090 | 1000 | 200 | 2000 | 55 | 10 | 67 |
| SMS+Straw Treatment-c T0 | 15000 | 3200 | 1200 | 240 | 2000 | 83 | 12 | 64 |
| SMS+Straw Treatment-a T1 | 13000 | 2800 | 1100 | 230 | 2200 | 78 | 9.6 | 40 |
| SMS+Straw Treatment-b T1 | 22000 | 1800 | 950 | 200 | 1600 | 77 | 8.7 | 35 |
| SMS+Straw Treatment-c T1 | 14000 | 3000 | 1300 | 270 | 2500 | 110 | 12 | 62 |
| Solvent blank 1 | 0.49 | 0.19 | 0.22 | 0.13 | 0.04 | 0.06 | 0.32 | 1.1 |
| Solvent blank 2 | 1.1 | 0.73 | 0.32 | 0.17 | 0.08 | 0.04 | 0.30 | 0.78 |
| Solvent blank 3 | 0.93 | 0.63 | 0.26 | 0.14 | 0.07 | 0.02 | 0.21 | 0.69 |
| Solvent blank 4 | 0.20 | 0.14 | 0.04 | 0.22 | 0.06 | 0.08 | 0.18 | 0.64 |
|  |  |  |  |  |  |  |  |  |
| **Average**  **(μg)** | *p.p'-DDT* | *o.p'-DDT* | *p.p'-DDD* | *o.p'-DDD* | *p.p'-DDE* | *p.p'-DDMU* | *p.p'-DDM* | *p.p'-DBP* |
| Starting Soil | 12000 | 3200 | 1200 | 260 | 2300 | 120 | 14 | 64 |
| Control T0 | 12000 | 2800 | 990 | 220 | 2000 | 130 | 11* | 52 |
| Control T1 | 12000 | 2800 | 900 | 200 | 1900 | 93 | 7.5* | 54 |
| Exp. blank T0 | 9500 | 2100 | 710 | 160 | 1400 | 81 | 5.8 | 43 |
| Exp. blank T1 | 9300 | 2200 | 960 | 230 | 1800 | 94 | 8.7* | 38 |
| SMS Treatment T0 | 11000 | 2400 | 750 | 170 | 1800 | 80 | 6.1* | 37 |
| SMS Treatment T1 | 12000 | 2300 | 1000 | 220 | 2000 | 92 | 8.0 | 41 |
| SMS+Straw Treatment T0 | 14000 | 3200 | 1130 | 220 | 2100 | 78 | 11 | 72 |
| SMS+Straw Treatment T1 | 16000 | 2500 | 1100 | 230 | 2100 | 88 | 10 | 45 |
|  |  |  |  |  |  |  |  |  |
| **Stdev**  **(μg)** | *p.p'*-DDT | *o.p'*-DDT | *p.p'*-DDD | *o.p'*-DDD | *p.p'*-DDE | *p.p'*-DDMU | *p.p'*-DDM | *p.p*'-DBP |
| Starting Soil | 1500 | 430 | 160 | 42 | 320 | 13 | 3.0 | 8.0 |
| Control T0 | 1400 | 300 | 140 | 25 | 180 | 22 | 0.9* | 2.1 |
| Control T1 | 1100 | 386 | 135 | 32 | 190 | 23 | 5.5* | 11 |
| Exp. blank T0 | 2000 | 480 | 170 | 43 | 300 | 16 | 4.7 | 17 |
| Exp. blank T1 | 1200 | 320 | 130 | 30 | 210 | 19 | 0.7* | 12 |
| SMS Treatment T0 | 2100 | 510 | 180 | 31 | 340 | 25 | 4.3* | 7.9 |
| SMS Treatment T1 | 3000 | 740 | 280 | 58 | 610 | 31 | 2.3 | 17 |
| SMS+Straw Treatment T0 | 3400 | 220 | 92 | 17 | 58 | 17 | 0.65 | 10 |
| SMS+Straw Treatment T1 | 3800 | 520 | 150 | 29 | 400 | 14 | 1.2 | 12 |

LOQ: Limit of quantification; <RL: < the recovery limit of 50%; *indicates one removed value from the triplicate measurement, i.e. *n*=2 in averages and standard deviation.

**Figure S3**: Estimated marginal means plot presenting individual DDX compound amounts (μg) before and after SMS mesocosm incubation, as predicted by emmeans modelling. (*n*=3)

**Table S9**: Theoretical and experimental soil concentrations (ng g^-1^) of DDT and its transformation products before (T0) treatment in the mesocosm study, based on starting soil concentrations and dry weight of soil and substrate. Standard deviation is shown for experimental data. Dicofol and *o,p'*-DDE data are missing due to too low recovery values.

| **Compound concentration (ng g^-1^)** | *p,p'*-DDT | *o,p'*-DDT | *p,p'*-DDD | *o,p'*-DDD | *p,p'*-DDE | *p,p'*-DDMU | *p,p'*-DDM | *p,p*'-DBP | Total |
| --- | --- | --- | --- | --- | --- | --- | --- | --- | --- |
| Starting soil | 2700±330 | 710±95 | 260±36 | 57±9 | 500±71 | 26±3 | 3.1±0.6 | 14±2 | 4200 |
|  |  |  |  |  |  |  |  |  |  |
| Exp. blank Theoretical | 2700 | 710 | 260 | 57 | 500 | 26 | 3.1 | 14 | 4200 |
| Exp. blank Experimental | 2500±520 | 560±130 | 190±45 | 43±11 | 380±78 | 21±4 | 2±0.8 | 11±4 | 3700 |
|  |  |  |  |  |  |  |  |  |  |
| Control Theoretical | 2400 | 650 | 240 | 53 | 460 | 24 | 2.9 | 13 | 3900 |
| Control Experimental | 3000±350 | 670±71 | 240±33 | 52±6 | 490±44 | 32±5 | 3.1±0.2 | 13±0.5 | 4500 |
|  |  |  |  |  |  |  |  |  |  |
| SMS Treatment Theoretical | 2500 | 650 | 240 | 53 | 460 | 24 | 2.9 | 13 | 3900 |
| SMS Treatment Experimental | 2500±490 | 590±120 | 180±43 | 39±7 | 420±82 | 19±6.1 | 2±0.1 | 9±2 | 3800 |
|  |  |  |  |  |  |  |  |  |  |
| SMS+Straw Treatment Theoretical | 2400 | 640 | 230 | 52 | 450 | 23 | 2.8 | 13 | 3800 |
| SMS+Straw Treatment Experimental | 4200±660 | 760±51 | 270±22 | 53±4 | 480±14 | 18±4 | 3.0±0.1 | 17±2 | 5800 |

**Table S10**: Soil concentrations (ng g^-1^ dw) of DDT and its transformation products before (T0) and after (T1) treatment in the field trial. Values below LOQ or outside the limits of recovery (<50% or >200%) were excluded from further evaluation.

| **Soil concentration**  **(ng g^-1^ dw)** | *p,p'*-DDT | *o,p'*-DDT | *p,p'*-DDD | *o,p'*-DDD | *p,p'*-DDE | *o,p'*-DDE | *p,p'*-DDMU | *p,p'*-DDM | *p,p*'-DBP | Dicofol |
| --- | --- | --- | --- | --- | --- | --- | --- | --- | --- | --- |
| *P. ostreatus* T0 plot-a | 2500 | 760 | 980 | 43 | 620 | 590 | 27 | 0.26 | 21 | 78 |
| *P. ostreatus* T0 plot-b | 3200 | 810 | 140 | 49 | 680 | 630 | 29 | 0.44 | 26 | 85 |
| *P. ostreatus* T0 plot-c | 3200 | 740 | 120 | 43 | 550 | 530 | 24 | 0.31 | 28 | 72 |
| *P. ostreatus* T1 plot-a | 5600 | 1400 | 180 | 55 | 970 | 860 | 39 | 1.9 | 41 | 110 |
| *P. ostreatus* T1 plot-b | 3600 | 790 | 130 | 44 | 590 | 560 | 28 | 0.56 | 30 | 75 |
| *P. ostreatus* T1 plot-c | 5100 | 1000 | 150 | 50 | 710 | 660 | 31 | 0.90 | 33 | 97 |
| *S. rugosannulata* T0 plot-a | 5600 | 830 | 150 | 51 | 690 | <LOQ | 24 | <LOQ | 27 | 94 |
| *S. rugosannulata* T0 plot-b | 4200 | 1000 | 170 | 56 | 790 | <LOQ | 31 | <LOQ | 34 | 110 |
| *S. rugosannulata* T0 plot-c | 3700 | 800 | 140 | 47 | 610 | 460 | 20 | <LOQ | 25 | 87 |
| *S. rugosannulata* T1 plot-a | 4400 | 920 | 170 | 53 | 690 | 500 | 26 | <LOQ | 31 | 95 |
| *S. rugosannulata* T1 plot-b | 4500 | 960 | 180 | 57 | 690 | 520 | 29 | <LOQ | 34 | 110 |
| *S. rugosannulata* T1 plot-c | 5200 | >RL | 180 | 56 | 770 | <LOQ | 28 | <LOQ | 35 | 110 |
| Solvent blank 1 | 1.8 | 1.2 | 0.45 | 0.35 | <LOD | <LOD | 0.066 | <LOD | 0.47 | 0.075 |
| Solvent blank 2 | 4.0 | 2.0 | 0.84 | 0.48 | <LOD | <LOD | 0.13 | <LOD | 1.0 | 0.14 |

LOQ: Limit of quantification; LOD: Limit of detection; >RL: < the recovery limit of 200%

**Table S11**: The concentration of DDX within the fruiting bodies of *P. ostreatus.* Values outside of recovery limits (50%-200%) were excluded from further evaluation and are not included in calculations of averages and standard deviation. *indicates a removed value, and where *n*=2 in averages and standard deviation.

| **Fruiting body DDX concentration (ng g^-1^ dw)** | *p,p'*-DDT | *o,p*'-DDT | *p,p'*-DDD | *o,p'*-DDD | *p,p'*-DDE | *o,p'*-DDE | *p,p'*-DDMU | *p,p'*-DDM | *p,p'*-DBP | Dicofol |
| --- | --- | --- | --- | --- | --- | --- | --- | --- | --- | --- |
| Plot 1 | 700 | 160* | 97 | 21 | 170 | <LOQ | 24 | 0.49 | 4.0 | 9.2 |
| Plot 2 | 710 | 140* | 65 | 14* | 94 | <LOQ | 20 | 0.46 | 4.4 | 13 |
| Plot 3 | 590 | 99* | 110 | 20 | 150 | <LOQ | 16 | 0.35 | 4.2 | 0.60* |
| Blank | 0.43 | 0.44 | 0.18 | 0.19 | 0.34 | 0.02 | 0.07 | 0.24 | 0.77 | 0.13 |
| **Average** | 661 | 130 | 90 | 20 | 137 | - | 20 | 0.43 | 4.2 | 7.5 |
| **Stdev** | 52 | 21 | 19 | 0.72 | 32 |  | 3.2 | 0.064 | 0.17 | 5.0 |
|  |  |  |  |  |  |  |  |  |  |  |

LOQ: Limit of quantification
